# Malignant epithelioid neoplasm of the ileum with ACTB-GLI1 fusion mimicking an adnexal mass

**DOI:** 10.1186/s12905-022-01679-0

**Published:** 2022-04-06

**Authors:** Ambrosio Marco, Virgilio Agnese, Raffone Antonio, Arena Alessandro, Raimondo Diego, Alletto Andrea, Seracchioli Renato, Casadio Paolo

**Affiliations:** 1 Mother-Child Department, Ospedale Maggiore, Azienda USL di Bologna, 40100 Bologna, Italy; 2 Division of Gynaecology and Human Reproduction Physiopathology, Department of Medical and Surgical Sciences, IRCCS Azienda Ospedaliero-Universitaria di Bologna, S. Orsola Hospital, University of Bologna, Via Massarenti 13, 40138 Bologna, Italy; 3 Gynecology and Obstetrics Unit, Department of Neuroscience, Reproductive Sciences and Dentistry, School of Medicine, University of Naples Federico II, Naples, Italy

**Keywords:** GLI1 fusion, Ultrasound, Epithelioid neoplasm, Case-report

## Abstract

**Background:**

Malignant epithelioid neoplasm with ACTB-GLI1 fusion are considered different from the more common pericytic lesions, such myopericytoma, because they have a spectrum of different genetic abnormalities. They appear to pursue a benign clinical course in young adults, although in sporadic cases lymph node metastasis were described. The categorization of this new type of tumor may also lead to new therapeutic strategies, because they might be sensitive to SHH pathway inhibitors.

**Case presentation:**

The case involves a 72-years-old multiparous woman who accessed our department after an incidental finding of a right adnexal mass of 43 mm with contrast-enhancement on a control computed tomography scan made for suspected diverticulitis. Our intervention was a detailed ultrasound description of the suspected neoplasm; a diagnostic laparoscopy and the contextual laparotomic removal of abdominal mass; its histological and immunohistochemical analysis. Our main outcome measure is the definition and future recognition of new pathologic entity called malignant epithelioid neoplasm with ACTB-GLI1 fusion.

**Conclusions:**

We described for the first time the ultrasound characteristic of this type of lesion using standardized terminology and we believe that it may be the first step to improve the acknowledgement of this novel pathologic entity defined as malignant epithelioid neoplasm with GLI-1 fusions.

**Supplementary information:**

The online version contains supplementary material available at 10.1186/s12905-022-01679-0.

## Background

GLI (glioma-associated oncogene homologue) expression in adult tissues is restricted to the fallopian tube, myometrium, and testis. GLI functions as an effector of the sonic hedgehog (SHH) pathway, inducing upregulation or downregulation of multiple downstream targets [1]. Shh signaling plays an essential role in embryonic development and it is critical for maintenance of tissue polarity. The Shh pathway is tightly regulated in most adult tissues but hyperactivation of this pathway is found in many solid tumors and aberrant Shh signaling has been implicated in many human cancers that account for up to 25% of human cancer deaths [2].

ACTB (beta-acting gene-glioma)-GLI1 fusions, resulting from a translocation (7;12) (p21–22, q13–15,) have been reported as the pathognomonic genetic abnormality described in rare characteristic mesenchymal neoplasm with a pericytic phenotype formed by monomorphic spindle cells and immunoreactivity for smooth muscle actin and laminin. These lesions described for the first time as “pericytoma with the t (7;12) translocation” are known as perivascular myoid tumors [3, 4]. They are considered different from the more common pericytic lesions, such myopericytoma or glomus tumor, because they have a spectrum of different genetic abnormalities [1].

The activation of GLI1 is made by a promoter change by the ubiquitously expressed ACTB, leading a deregulation of GL1-1 expression and its downstream targets. A combined approach including RNA-sequencing, target exome sequencing and FISH analysis were used to identify these new genetic abnormalities [5–7].

Most of these tumors were found in the soft tissue and in the tongue, with rare cases detected in the stomach and bone. Given the small number of cases. it is difficult to determine the average age of onset; however, most patient were younger than 40 years at diagnosis [1]. They appear to pursue a benign clinical course in young adults, although in sporadic cases lymph node metastasis were described. In cases where lymph node metastases were described patients were still alive after a mean follow up of 50 months [1].

In accordance with precision medicine principles, a more and more tailored approach is advisable to improve survival in oncological patients, highlighting the need for a precise diagnosis for each lesion [8]. The categorization of this new type of tumor may lead to new therapeutic strategies, because they might be sensitive to SHH pathway inhibitors [2].

We present a rare case of malignant epithelioid neoplasm of the ileum with ACTB-GLI1 fusion mimicking an adnexal mass.

## Case presentation

We present a case of a 72-years-old multiparous woman who accessed our Gynecology and Human Reproduction Physiopathology Unit in September 2020 after an incidental finding of a right adnexal mass of 43 mm with contrast-enhancement on a control computed tomography (CT) scan made for diverticulitis in the department of Gastroenterology of a first-level hospital. Tumoral markers, including CEA, CA 19-9, CA 15-3 and CA 125 were negative.

The patient had a history of arterial hypertension, glaucoma and diverticulitis. Moreover, in her 40s she underwent laparotomic cholecystectomy surgery.

The diagnostic work-up and the surgical management are shown in Additional file 1: Video.

**Fig. 1:**
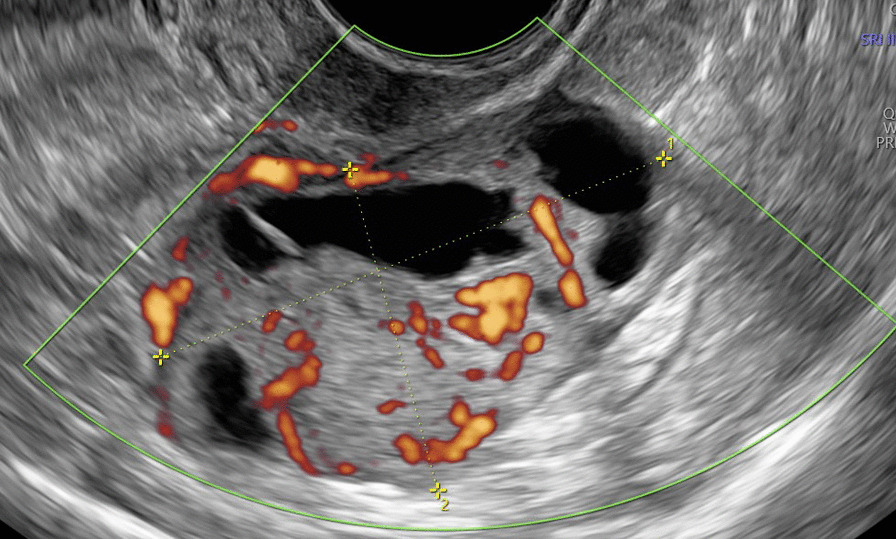
Power Doppler transvaginal ultrasound image of a solid inhomogeneous mass (solid component > 80%) with multiple anechoic cysts, irregular external contour and with a remarkable vascularization at Power Doppler (Color Score 4)

**Fig. 2:**
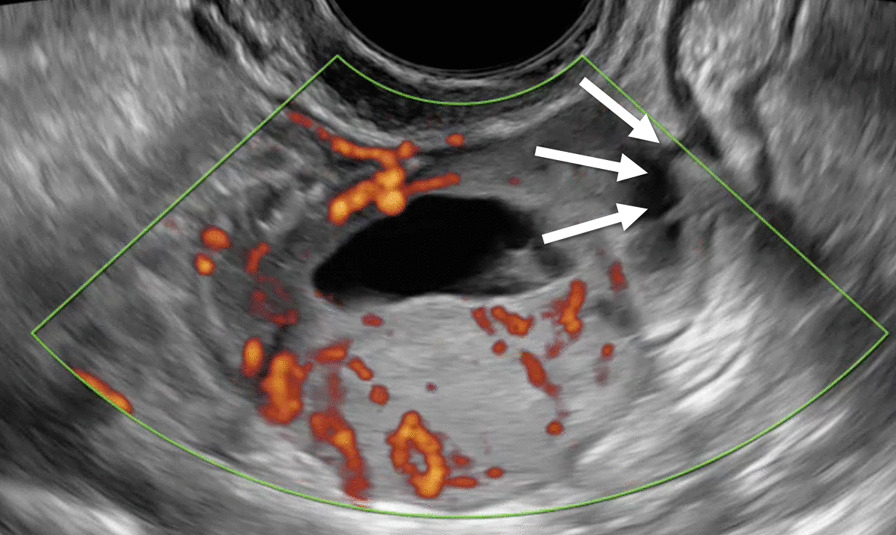
Power Doppler ultrasound image of the possible origin of the tumor

At the admission in our department, she was asymptomatic and a transvaginal (TV) and a transabdominal (TA) ultrasound (US) were carried out by an expert sonographer with at least 5 years of experience in onco-gynecological ultrasound. US showed a normal anteverted uterus with normal left adnexa. A solid tumor that measured 44 × 22 × 43 mm was identified adjacent to the right ovary. According to International Ovarian Tumor Analysis (IOTA) terminology [9] it was a solid inhomogeneous mass (solid component > 80%) with multiple anechoic cysts, irregular external contour and with a remarkable vascularization at Power Doppler (Color Score 4) (Fig. 1). The sliding tumor sign was present. The origin of the tumor was unclear; however, the position and the presence of the sliding sign with the right ovary seemed to indicate an intestinal origin (Fig. 2). No free fluid was seen in the Douglas pouch. Visible peritoneal implants, omental thickenings, and hepatic, splenic and lymph node secondary involvement were not observed. The examiner classified this tumor as probably malignant and the suspect diagnosis according to the literature was Extra Gastrointestinal Stromal tumor (eGIST) [10]. A tumor staging CT scan was carried out in our department that was negative for infiltrated lymph nodes and distant metastasis.

**Fig. 3:**
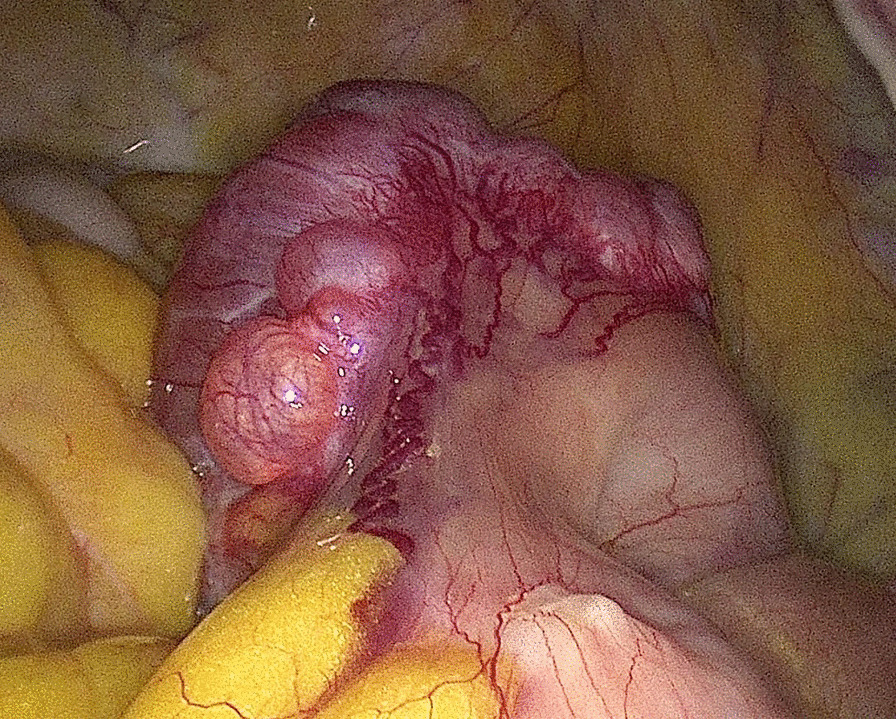
Laparoscopic view of the tumor

**Fig. 4:**
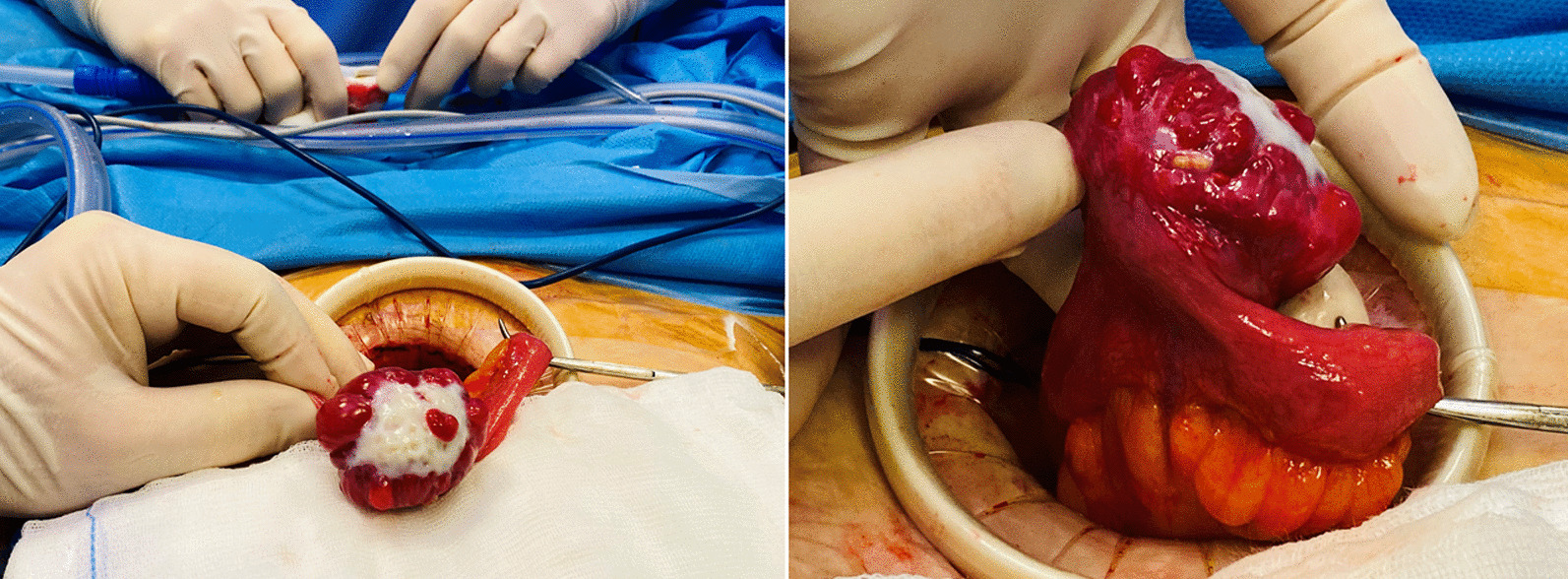
Laparotomic view of the tumor rising form an ileal loop

The patient was then scheduled for a diagnostic laparoscopy that showed normal uterus and normal adnexa. An exophytic cerebriform whitish tumor of about 4.5 cm, rising form an ileal loop was observed (Fig. 3). Laparotomic removal of the ileal tumor by segmental intestinal resection and latero-lateral anastomosis was performed (Fig. 4) and the tissue sent for histopathologic examination.

The post-operative course was regular, and the patient was discharged from our ward without any complications.

After some difficulties to identify the histological nature of the tumor, the samples from the ileal lesion were sent to a referral center for further analysis. They confirmed the nature of a malignant mural-based neoplasm. The slides showed a nested epithelioid malignancy that was located within the muscularis propria and the submucosa. The neoplastic cells were uniform and had a clear, vacuolated cytoplasm, small round nucleoli with fine chromatin, and focally prominent nucleoli. ln some areas, the tumor exhibited a moderate amount of hyalinized stroma with a capillary network. This stroma was minimal at the base of the tumor.

**Fig. 5:**
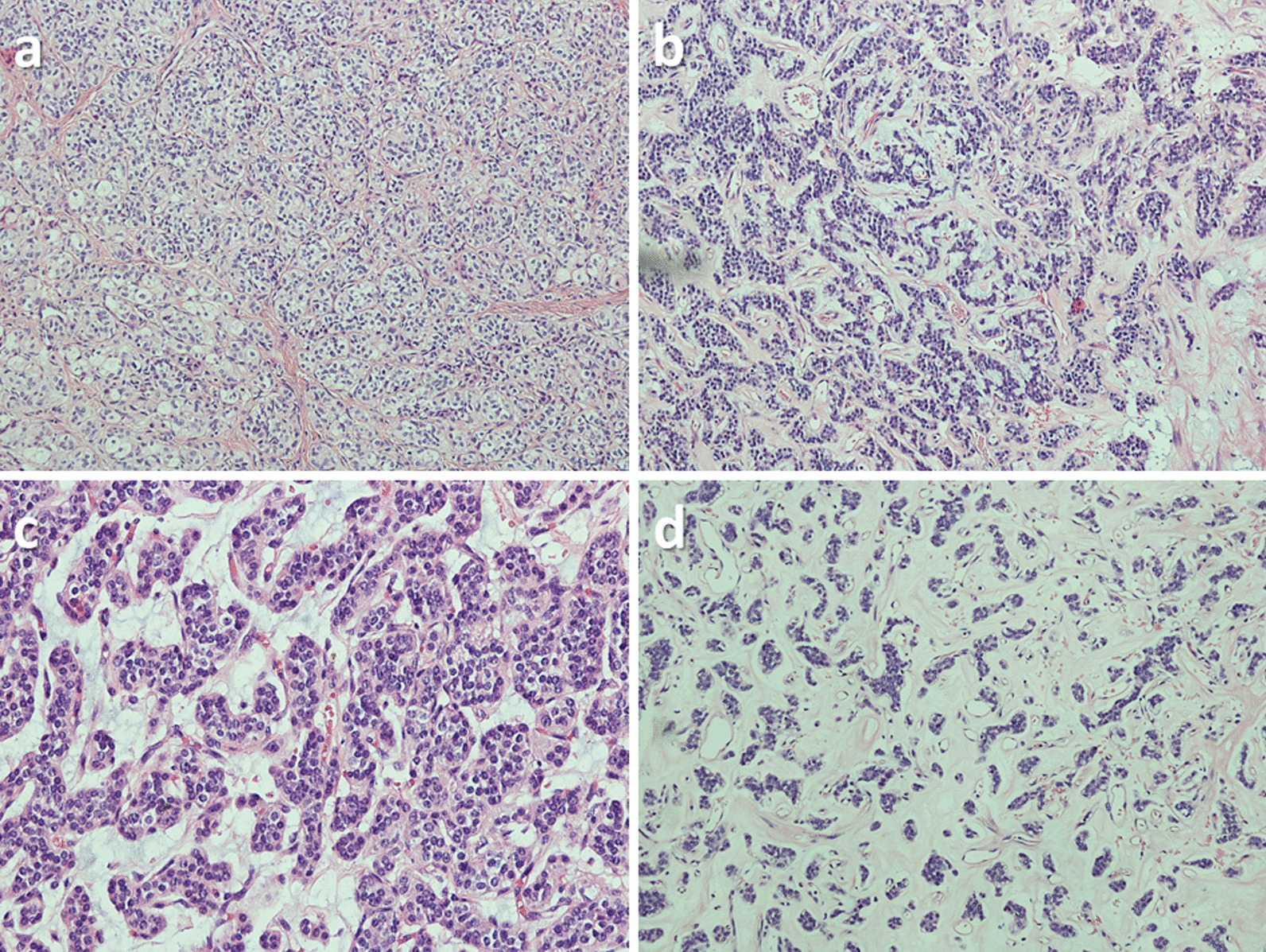
Cellular morphology of the tumor: **a** solid component characterized by nested epithelioid clear cells with thin interlayer stroma (haematoxylin and eosin, ×10); **b** Ridges of epithelioid cells with myxoid stroma (haematoxylin and eosin, ×10); **c** solid component characterized by nested epithelioid clear cells with thin interlayer stroma (haematoxylin and eosin, ×20); **d** epithelioid cells dispersed in myxoid stroma (haematoxylin and eosin, ×10)

Immunohistochemical stains showed expression of vimentin, CD10, p63, CD56, and cyclin D1, but did not express CK7l18/8, EMA, BERP4, synaptophysin, chromogranin, calretinin, inhibin, PAX8, WT1, ER, PR, SMA, desmin, caldesmin, CD34, Ckit, DOG1, HMB45, and MART1. Additional immunohistochemical stains showed that the neoplastic cells express S100 in the areas with decreased stroma but did not express SOX10. An immunohistochemical stain for TFE3 was equivocal, the differential diagnosis would include several mesenchymal neoplasms, including clear cell sarcoma-like tumor of the gastrointestinal (GI) tract, which can share histologic features along with S100 labeling; however, the negative staining for SOX10 argued against that possibility. They also considered the recently described entity of soft tissue sarcoma with GLI1 fusions. GLI1 fusion analysis by both FISH and ARCHER were conducted. Both tests founded an in-frame ACTB-GLI1 fusion involving exon 2 of ACTB and exon 6 of GLI1. Given the histomorphology and the fusion identified the final diagnosis was malignant epithelioid neoplasm with GLI1 fusion (Fig. 5).

After a multidisciplinary meeting, involving the oncologists, the surgeons, the pathologists and the radiologists, and in consideration of the initial stage of the pathology, there were no indications for further therapies. Moreover, because the lack of a defined oncological follow up program, the patient was scheduled for annual follow up.

## Discussion and conclusions

We showed a rare case of malignant epithelioid neoplasm of the ileum with ACTB-GLI1 fusion mimicking an adnexal mass. The differential diagnosis of this type of tumor may be challenging, especially when the ovaries are not easily recognizable.

ACTB-GLI1 fusion was first described in 2004 by Dahlen and co-workers [3, 4] in a pericytoma with t (7;12) translocation, a rare soft tissue neoplasm associated with a benign clinical course and pericytic differentiation based on its smooth muscle actin (SMA)-positive/S100-negative immunophenotype and ultrastructural characteristics. However, several recent studies have demonstrated GLI1 genetic abnormalities in a group of soft tissue tumors with shared morphologic features but a more variable immune-profile, which often includes S100 positivity, and propensity for malignant behavior, including regional and/or distant metastasis [1, 5, 11–15].

The genetic alterations encountered in these tumors included not only GLI1 fusions with ACTB gene but also with other partners, such as MALAT1 and PTCH1 as well as high-level GLI1 gene amplifications [15].

Xu et al. classified all the 35 cases of mesenchymal neoplasms with GLI1 alterations reported to date [1, 3–5, 11–15]. Nineteen cases presented ACTB-GLI1 fusions, and they originated from different location (i.e. tongue, neck, ovary, retroperitoneum), among them two cases developed distant metastasis and other two cases developed regional recurrences. After an average 4-years follow up there were no evidence of recurrent disease [15]. Antonescu et al. also described two cases with PTCH1-GLI1 fusions and other two cases with MALAT1-GLI1 fusions. One patient with PTCH1-GLI1 fusions developed lung metastasis. In every patient there were no evidence of recurrent disease [1].

Xu et al. described a cohort of 11 head and neck lesions with GLI1 alteration, including 8 from the tongue. Two out of six patient with available follow up developed local recurrence and distant metastases, at 6 and 83 months, respectively [15].

The peculiar immune-profile and behavior of this group of malignant mesenchymal neoplasm has allowed to define a novel pathologic entity called malignant epithelioid neoplasm with GLI1 fusions [1].

Identifying GLI1 alteration and oncogenic activation in this unique tumor entity might allow the patients, especially those with distant metastasis or not fit for surgery, to gain access to targeted therapies [15]. Particularly, some inhibitors targeting the hedgehog pathway, including GLI inhibitors (i.e. arsenic trioxide, pirfenidone, and imiquimod) are currently approved by the US Food and Drug Administration (FDA) or are available in various clinical trials to treat leukemia, basal cell carcinoma, and other types of carcinoma[2, 15].The Sonic Hedgehog (Shh) signaling pathway is a major regulator of cell differentiation, cell proliferation, and tissue polarity. Aberrant activation of the Shh pathway has been shown in a variety of human cancers (i.e. basal cell carcinoma, malignant gliomas, medulloblastoma) and the downstream effectors of the Shh pathway including smoothened (SMO) and glioma-associated oncogene homolog (GLI) family of zinc finger transcription factors are regarded as important targets for cancer therapeutics [2]. The categorization of this new type of tumor might lead to new therapeutic strategies, because they might be sensitive to SHH pathway inhibitors [1].

We described for the first time the ultrasound characteristic of this type of lesion using standardized terminology and we believe that it may be the first step to improve the acknowledgement of this novel pathologic entity defined as malignant epithelioid neoplasm with GLI-1 fusions [1].

While CT misled the diagnosis of this tumor, ultrasound helped to identify the correct origin and the malignant nature of the lesion improving the preoperative work-up. Moreover, transvaginal ultrasound allows a better characterization of the mobility of the tumor trough the sliding organ sign [16] and in expert hands may provide crucial information to correctly manage the lesion [17]. Particularly IOTA rules were applicable even in the case of a non-adnexal lesion, indicating malignancy.

**Fig. 6:**
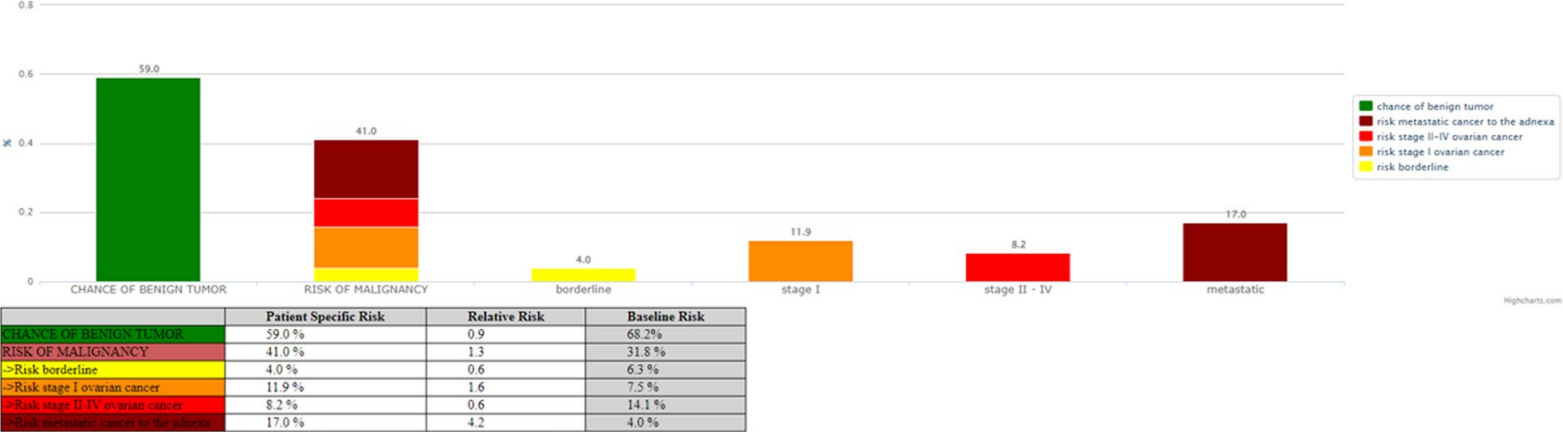
Analysis of the tumor performed with the ADNEX model

IOTA terminology and the subsequent models such us the ADNEX model [9, 18] has been a breakthrough in ultrasound diagnosis for adnexal tumors. The IOTA group showed that polytomous risk prediction for the diagnosis of ovarian cancer is feasible [18], however these systems may be further improved [19, 20]. For research purpose we applied retrospectively the ADNEX model on this tumor and the findings were consistent to the subjective evaluation of the original examiner (Fig. 6) if a 10% cut-off is used. However, it might be useful to understand why the percentage of benignity, from the ADNEX model, is so elevated in this kind of tumors.

The acknowledgement of specific ultrasound features (i.e. irregular margins, abundant vascularization, cystic areas) allows a better characterization of malignant tumors [21] and may lead to more tailored approach.

In conclusion we believe that ultrasound may help to characterize a new pathologic entity, defined as malignant epithelioid neoplasm with GLI-1 fusions even better than CT. The IOTA terminology may be useful to suspect malignant lesions even in the case of this rare tumor.

## Supplementary Information

**Additional file 1: Video**. Detailed ultrasound description of the suspected neoplasm. Diagnostic laparoscopy and laparotomic removal of abdominal mass. Histological and immunohistochemical examination.

## Data Availability

Not applicable.

## Abbreviations

GLI: Glioma-associated Oncogene Homologue
SHH: Sonic Hedgehog
ACTB: Beta-acting gene-glioma
CT: Computed Tomography
TV: Trans-vaginal
TA: Trans-abdominal
US: Ultrasound
IOTA: International Ovarian Tumor Analysis
eGIST: Extra Gastrointestinal Stromal tumor
GI: Gastrointestinal
SMA: Smooth Muscle Actin
