# Correction to: Malignant epithelioid neoplasm of the ileum with ACTB-GLI1 fusion mimicking an adnexal mass

**DOI:** 10.1186/s12905-022-01766-2

**Published:** 2022-05-25

**Authors:** Marco Ambrosio, Agnese Virgilio, Antonio Raffone, Alessandro Arena, Diego Raimondo, Andrea Alletto, Renato Seracchioli, Paolo Casadio

**Affiliations:** 1 Mother‑Child Department, Ospedale Maggiore, Azienda USL Di Bologna, 40100 Bologna, Italy; 2 Division of Gynaecology and Human Reproduction Physiopathology, Department of Medical and Surgical Sciences, IRCCS Azienda Ospedaliero‑Universitaria di Bologna, S. Orsola Hospital, University of Bologna, Via Massarenti 13, 40138 Bologna, Italy; 3 Gynecology and Obstetrics Unit, Department of Neuroscience, Reproductive Sciences and Dentistry, School of Medicine, University of Naples Federico II, Naples, Italy

## Correction to: BMC Women's Health (2022) 22:104 10.1186/s12905-022-01679-0

Following publication of the original article (1), The author names were incorrectly published as Ambrosio Marco, Virgilio Agnese, Raffone Antonio, Arena Alessandro, Raimondo Diego, Alletto Andrea, Seracchioli Renato and Casadio Paolo. But this should have been Marco Ambrosio, Agnese Virgilio, Antonio Raffone, Alessandro Arena, Diego Raimondo, Andrea Alletto, Renato Seracchioli, and Paolo Casadio.

The original article has been updated.

